# Evolving Ablative Therapies for Hepatic Malignancy

**DOI:** 10.1155/2014/230174

**Published:** 2014-04-29

**Authors:** Smit Singla, Steven N. Hochwald, Boris Kuvshinoff

**Affiliations:** Roswell Park Cancer Institute, Elm and Carlton Street, Buffalo, NY 14263, USA

## Abstract

The liver is a common site for both primary and secondary malignancy. Hepatic resection and transplantation are the two treatment modalities that have been shown to achieve complete cure, but only 10 to 20% of patients are candidates for these treatments. For the remaining patients, tumor ablation has emerged as the most promising alternative modality. In addition to providing local control and improving survival outcomes, tumor ablation also helps to down stage patients for potential curative treatments, both alone as well as in combination with other treatments. While tumor ablation can be achieved in multiple ways, the introduction of newer ablative techniques has shifted the focus from palliation to potentially curative treatment. Because the long-term safety and survival benefits are not substantive at present, it is important that we strive to evaluate the results from these studies using appropriate comparative outcome methodologies.

## 1. Introduction

The liver is a common site for both primary as well as secondary metastatic tumors and a small of these patients are considered candidates for liver-directed therapies. Hepatocellular carcinoma (HCC) is the most common primary tumor, with an estimated 30,640 new cases and 21,670 deaths in 2013 in the USA [1]. Worldwide, HCC is recognized as the most common solid tumor and third leading cause of cancer-related deaths [2, 3]. The liver is also the site for metastatic lesions from other sources including colon and rectum. Amongst more than 150,000 new cases of colorectal cancer diagnosed annually in the USA, nearly 1/3 of patients present with synchronous metastatic lesions and another 2/3 develop metachronous lesions to the liver [4]. Similar to colon cancer, patients with breast, ovary, kidney, and neuroendocrine tumors also metastasize to the liver and present with secondary metastatic lesions.

Traditionally, hepatic resection and to an extent hepatic transplantation are considered the only definitive treatment modalities capable of achieving cure. However, only 10 to 20% of patients qualify or are considered candidates for these treatments [5]. For the remaining patients, there has been a sustained effort to develop alternate treatment modalities. To that end, amongst the various liver-directed regional therapies that have been developed in the last decade, tumor ablation (TA) has been shown to be the most promising. TA not only allows local control, but it also helps to down stage patients, which can then let some of these patients become qualified for potential curative treatments.

TA can be achieved by utilizing a wide array of techniques and technology including percutaneous chemical injection using ethanol (PEI) or acetic acid (PAI), cryoablation (CA), radiofrequency ablation (RFA), laser-induced thermotherapy (LITT), high-intensity focused ultrasound (HIFU), microwave ablation or coagulation (MWA), and irreversible electroporation (IRE) (Table 1). While every technique is inherently different and has its own advantages and disadvantages, MWA and IRE have been shown recently to offer newer alternative as compared to their counterparts [6]. In this paper, we provide a review of different ablation techniques with emphasis on MWA and IRE in treatment of liver malignancy and its outcome.

TA has evolved significantly since its inception many years ago. Developed originally as a means to provide alternative option for patients with unresectable tumors, TA now has a broad range of indications including both primary therapy and in conjunction with other treatment modalities. This has been made possible due to multiple studies that suggested improved outcomes in patients treated with TA in the last few years [7–9].

For patients with HCC, if left untreated, the 5-year survival rates are less than 20% [7]. On the other hand, for patients who are candidates, studies have shown that the best 5-year survival rates are achieved with hepatic resection or transplantation [7, 10]. However, as we know, not all patients are candidates for either of these treatment modalities for a variety of reasons. These include limited number of donor livers, presence of tumors in unresectable locations, too many tumors, inadequate hepatic reserve, or presence of multiple comorbidities [7, 10].

In patients with colorectal metastatic lesions, multiple studies have shown significant improvement in survival with surgical resection. In early series, Wilson and Adson [11] and Attiyeh et al. [12] reported nearly 40% of 5-year survival and 20% of 10-year survival. With increased experience and appropriate selection, recent studies have published 5-year survival rates in excess of 50% [13–17]. However, despite significant advances, less than 25% of patients with hepatic metastases are eligible for surgical resection [18]. If we therefore combine all patients with primary and secondary hepatic malignancy, we find that only a small percentage of patients are eligible for hepatic resection or transplantation. This leaves a large subgroup of patients who, if left untreated, will invariably have poor prognosis and minimal survival. It is in these patients where TA has emerged as a viable treatment option and may demonstrate significant improvement in survival and outcome. The most up-to-date literature supports the use of TA in improving five-year survival in such patients. Recently, Itoh et al. [19] showed that MWA is effective and provides five-year survival rate of 43.1% in patients with unresectable HCC. Similarly, studies involving other TA techniques including RFA [20], CA [21], PEI [22] LITT [23], and HIFU [24] have been shown to achieve increased survival rates of 47.8% at 5 years, 24% at 5 years, 49% at 5 years, 33% at 5 years, and 62% at 3 years, respectively, in specific subsets of patients. Since IRE [25] is a relatively newer nonthermal technology, only a limited number of studies are available for review. Cheung et al. [25] recently showed that IRE use was associated with a local recurrence-free period of approximately 18 months and a distance recurrence-free period of about 14 months, suggesting that IRE is a safe and feasible technique for local ablation of hepatic lesions.

## 2. Ablation Techniques

### 2.1. Chemical Ablation

Percutaneous chemical ablation has worldwide acceptance for small hepatic tumors due to relative ease of use and reduced cost. Both ethanol (95%) and acetic acid have been utilized in ablation of small hepatic tumors; however, multiple studies have failed to show that one is better than the other [26]. PCI is performed generally as an outpatient procedure under ultrasound or computed tomography guidance, using either a narrow gauge needle or the newer Quadra-Fuse multiport needle device (Rex Medical, Conshohocken, PA). The basic principle is injection of the desired chemical (ethanol or acetic acid) to achieve chemical necrosis within the tumor. The resultant fibrotic reaction leads to microvasculature thrombosis and tumor ischemia [27]. However, to achieve clinically desired results, this process has to be repeated multiple times and over multiple sessions.

PEI remained very popular for ablation of nonresectable small hepatic tumors until the introduction of RFA. Subsequently, multiple studies compared PEI with RFA, and found that RFA was associated with better outcomes [28]. In a recent large meta-analysis, Orlando et al. [29] showed that RFA was superior to PEI with respect to tumor response, risk of local recurrence, and 3-year cancer-free survival as well as overall survival in patients with small sized HCC. PEI has also been compared to surgical resection. Chen conducted a randomized controlled trial comparing PEI and surgical resection in 160 patients with a solitary HCC up to 5 cm in diameter. The study did not find any significant difference in either disease-free or overall survival [30].

PEI has also been evaluated by combining it with other treatments such as transarterial chemoembolization (TACE). In the a subset of patients with <2 cm HCC lesions, Koda et al. [31] found that a combination therapy of TACE + PEI was superior to PEI alone. The combined treatment resulted in a significantly lower cumulative detection rate of local residual disease when compared with the detection rates in the PEI alone group. While PEI was found to be helpful in a subset of patients with small sized HCC, it has performed poorly in patients with metastatic lesions. Giovannini [32] performed a comprehensive review evaluating the role of PEI in treating hepatic metastatic lesions. It was noted that PEI was effective in more than 50% of patients with hepatic lesions <4 cm in size; however, on detailed analysis, these results were applicable in only a selected group of patients. Further, PEI had to be utilized multiple times to achieve the desired results. When these results were compared to the results from other emerging ablation techniques available at that time (laser photocoagulation, CA, RFA and MWA), it was projected that PEI would be soon replaced by these techniques. With time, as more and more data from the newer techniques became available, PEI was found to be less useful. As a result, PCI is now sparingly used is the USA.  Table 2 provides a summary of studies investigating the role of chemical ablation in patients with hepatic malignancy.

### 2.2. Cryoablation

Nitrous oxide, liquid nitrogen, and argon have been used as cryogens for the last 50 years. In the last decade, however, there has been significant improvement in the technology, which has led to development of devices that allow delivery of cryogens using percutaneous insulated cryoprobes (Endocare, Inc., Irvine, CA and Galil Medical, Yokneam, Israel). These newer devices permit circulation of argon or liquid nitrogen such that the desired cooling temperature is achieved within seconds. This freezing causes formation of “ice ball,” within which the irreversible tissue destruction takes place and ablation ensues. A single probe can create an ablation zone of approximately 3 cm in diameter; however, using multiple probes, this size can be increased to >8 cm in diameter. These ablation zones are readily visible on imaging including ultrasound, CT scan, and MRI to help discern response to treatment.

CA has been traditionally used when liver tumors are close to vital structures like bile duct or blood vessels or when margins are close and in places where heat sink affect is anticipated. CA has seldom been used alone, and most of the data reported has been in combination with other methods. In a large series, Zhou and Tang [33] reported data on 235 patients where 78 patients were solely treated with cryotherapy, while the other patients received combination therapy including hepatic artery ligation, hepatic arterial infusion chemotherapy, TACE, and resection of the frozen tumor. They found that the 1-, 3-, and 5-year survival rates were similar in both groups, that is, patients undergoing cryotherapy alone (80%, 52%, and 40%, resp.) and patients receiving combined therapy (78%, 54%, and 40%, resp.). Another report by Wren et al. [34] looked at 12 patients with HCC who were treated with CA. Some of these patients were treated with intention to treat, while others received palliative intervention. It was found that, although CA was safe overall, it was more effective as a tool for palliation than definitive or curative treatment.

Haddad et al. [35] evaluated 31 patients with advanced hepatic tumors using CA in conjunction with surgical resection. Amongst 32 procedures, CA was used for primary ablation in 21 and as an adjunct in the remaining 11 procedures. All patients in whom CA was used as adjunct had close margins on pathology. The reported mortality and morbidity rates in this study were high at 6 and 60%, respectively. At the end of the study, the actuarial patient survival rates were found to be less impressive—90% and 22% at 6 and 36 months, respectively. Similarly, in patients with metastatic lesions to the liver, Seifert et al. [36] and Xu et al. [37] found that using CA alone was associated with higher local hepatic recurrence rates. They concluded that CA served well as a complement to surgical resection. It was beneficial as an additional means of achieving tumor eradication when total excision was not possible.

As the role of CA in the treatment of hepatic malignancy evolved, many studies began to raise concerns regarding serious side effects associated with this technology. CA was found to be associated with complications including cryoshock, hypothermia, cracking of the ice ball, hemorrhage, biloma, abscess, pleural effusion, and death [38, 39]. Because of relatively higher incidence of morbidity and mortality, reduced clinical efficacy, and inferior ease of use, CA was slowly replaced with thermal ablation techniques. It is now used very sparingly, in the setting of research purposes mostly.  Table 3 provides a summary of studies investigating the role of cryoablation in patients with hepatic malignancy.

### 2.3. Thermal Ablation

#### 2.3.1. Radiofrequency Ablation

In contrast to cryoablation, tissue ablation using thermal techniques have been well received worldwide. In addition to the ease of use, better safety profile and comparatively low cost, thermal techniques such as RFA and MWA have consistently shown better outcomes in multiple studies worldwide. TA is based on the principle that heat produces predictable tissue response with rise in temperature. The normal body begins to show signs of cellular damage after 45°C. Between 50–55°C, heat causes irreversible cellular damage, and, at temperatures between 60–100°C, heat can cause immediate coagulative necrosis. At any temperature above 100°C, heating of tissues leads to tissue vaporization [40].

Currently, RFA is the most widely used thermal ablation technique used worldwide for the treatment of liver tumors. RFA is based on the principle of generating heat within the tissue using high frequency alternating current (460–480 kHz) delivered via electrode(s). This alternating current causes agitation of ions, which generates frictional heat. As the temperature rises above 60°C, cells disintegrate and create a zone of necrosis around the electrode. A number of commercial devices including expandable multitined needle electrodes (AngioDynamics and Boston Scientific) or the internally cooled electrodes (Valleylab/Covidien) are available in the market. These devices are designed to create ablation diameters up to 4-5 cm and have been shown to have equivalent therapeutic effectiveness in patients with HCC up to 3 cm in diameter [40].

RFA has been typically utilized in patients with HCC who do not meet the criteria for surgical resection. While some studies have restricted RFA to subset of cirrhotic patients, others have shown benefits comparable to surgical resection [40, 41]. Additionally, RFA has also shown to be beneficial in treating patients with recurrent HCC in the liver following partial hepatectomy [42]. To clarify some of these questions, there have been controlled studies comparing RFA with surgical resection in patients with HCC. In the first study, 112 patients with a solitary HCC ≤ 5 cm were randomly assigned to surgical resection or percutaneous RFA [43]. When analyzed, the overall and disease-free survival rates were comparable in the two groups at one, two, and three years. In a similar fashion, a second trial randomly assigned 105 patients with a single HCC ≤ 5 cm or three or fewer lesions, all ≤3 cm, to percutaneous RFA or surgical resection [44]. The authors found that there were no local recurrences in either group, and the three-year overall survival rates were similar in both the groups (87 versus 86 percent for RFA and surgery, resp.).

However, in 2009, Ueno et al. [45], presented data from their large database of 278 consecutive patients with HCC, who met the Milan criteria. These patients were grouped into three groups: curative hepatic resection (*n* = 123), initial percutaneous RFA (*n* = 110), or surgical RFA (thoracoscopic, laparoscopic, or open; *n* = 45). On analysis, the authors found that, in patients meeting the Milan criteria, hepatic resection provided better outcomes in patients with single tumor and preserved liver function. They noted that RFA was better in patients with unresectable solitary tumors and those with multinodular tumors, regardless of the grade of liver damage. Further, surgical RFA provided increased long-term oncological control when compared to percutaneous RFA. To further evaluate patients meeting the Milan criteria, Huang et al. [46] organized a large randomized controlled trial with 230 patients. These patients were randomly assigned to surgical resection or RFA. The trial revealed that both the overall survival as well as the recurrence free survival were significantly better in the resection group as compared to the RFA group (76 versus 55% at 5 years and 51 versus 29%, resp.). Further, overall recurrence rates were also significantly lower with resection compared to RFA (42 versus 63%, resp.).

Given the lack of clear-cut guidelines regarding their usage, most clinicians prefer to utilize RFA in patients who are not ideal candidates for surgical resection. There is, however, one area where RFA has found increased acceptability. In patients awaiting transplant who progress or patients who can be “down-staged” to meet the transplant criteria, RFA is being increasingly utilized to “bridge” the gap [47]. Some authors have also investigated the use of RFA in the treatment of limited hepatic metastatic disease in patients not suitable for hepatic resection.

Mulier et al. [48] performed an exhaustive review of RFA for colorectal metastases. The authors reported that RFA was associated with worse local control, worse staging, and a small risk of electrode track seeding when compared with resection. For tumors less than 3 cm, local control after surgical RFA was equivalent to resection. Gillams and Lees [49] performed a multivariate analysis of 5-year survival in 309 patients with colorectal hepatic metastases, treated at 617 sessions using percutaneous RFA. They found that in selective patients, their five-year survival reached 24–33% after ablation, which was superior to any published chemotherapy data and approached the results of liver resection. In 2009, the American Society of Clinical Oncology published a review on utilization of RFA in colorectal metastasis [50]. This review supported the use of RFA as an adjunct to surgical resection in some patients but stopped short of providing definite guidelines. Instead, it proposed further research to better define the role of RFA in the treatment of hepatic malignancy. A recent randomized controlled trial showed that RFA combined with systemic treatment resulted in significant longer progression-free survival. This study, however, failed to clearly define the effect of RFA on overall survival [51]. Further, a recent systematic review also concluded that the evidence supporting the use of RFA in colorectal metastatic disease is currently insufficient and recommends further research before RFA becomes standard of care in treating patients with metastatic colorectal disease [52].

#### 2.3.2. Laser Interstitial Thermotherapy

LITT is another ablative technique that has been utilized for some years in patients with hepatic tumors. It uses an Nd-YAG (neodymium: yttrium aluminum garnet) diode laser to deliver a precise amount of energy at a defined location. It works by generating a characteristic monochromatic light at a wavelength of 1,064 nm for about 2–20 minutes to achieve a “therapeutic window.” During this period, based on the property of the tissue being ablated, the laser delivers photons, which generates heat and leads to the development of thermal coagulation at that site. As compared to other lasers, LITT produces slow heating within its therapeutic window. This allows maximal tissue penetration, and, therefore, desired therapeutic results.

This technology requires insertion of specific probes containing laser fibers into the tumor tissue. It typically produces a thermal effect between 10 and 20 mm in diameter. Due to its limitations in generating large ablation zones, this technology has found only a limited commercial success. The earlier series reported successful results; however, these series were small and lacked long-term data. Currently, there are only a few studies that have published long-term data. In 2001, Mack et al. [53] published a large series with long-term results in 705 patients with hepatic metastatic lesions. A total of 1981 lesions were treated over 1653 treatment sessions. The reported complication rate was 7.5% and the local tumor control rate was 97.9% at 6 months. Their 1- and 5-year survival rates were 93% and 50%, respectively. Another study in 2007 by Eickmeyer et al. [54] reported favorable results in 66 patients with nonresectable colorectal metastatic lesions. In their study, the authors found that their new internally water-cooled devices were safe and yielded large ablation zones with diameters ranging from 20 to 40 mm. The complication rate was 2.1% and the periprocedural mortality rate was 3%. After 12 months, local tumor control was 69.4%. Additionally, no metastatic deposits were detected along the catheter access route. A similar study by Eickmeyer et al. [54] found that the survival in patients treated with LITT was directly associated with user experience. Amongst 85 patients, they found that as their experience improved, the survival improved as well. Their 1- and 3-year survival rates were 93% and 56%, respectively.

A recently published randomized trial looked at the difference in survival in patients with hepatic lesions treated with transarterial oily chemoembolization combined with interstitial laser thermotherapy (TOCE + ILT) versus TOCE + PEI. The authors found that the 2-year survival rate was significantly higher in patients with TOCE + ILT (79.6% versus 60.8%) [55]. Further, another recent study published long-term survival and progression-free survival (PFS) in 594 patients with colorectal liver metastases treated with LITT. The study utilized a newer laser technology and found that their long-term survival and PFS rates were better—78 and 7.8% and 51.3 and 22.3% at 1- and 5-years, respectively [23].

Despite these promising results and introduction of newer devices that create large ablation zones, clinical acceptance by physicians remains low for this technology at present. It is hoped that as the technology advances and more robust data and improved outcomes become available, this technology will become more acceptable.

#### 2.3.3. High-Intensity Focused Ultrasound

HIFU or focused ultrasound is a subtype of thermal ablation technique that uses high-intensity ultrasound energy to locally heat and ablate tissue. This technology uses transducers that deliver high-intensity ultrasound in the range of 100–10,000 W/cm^2^ to a focal region. The absorption of this highly focused and intense acoustic energy leads to generation of temperatures above 60°C in a short interval of time. This leads to development of coagulation necrosis in the area of focus.

The clinical application of HIFU has seen slowly increasing and now includes treatment of both benign and malignant solid tumors. Many studies have shown successful ablation of hepatic tumors including HCC and metastatic hepatic lesions. The most recent data has been especially promising. Ng et al. [24] presented data on 49 patients who received HIFU for unresectable HCC. Majority of their patients (83.6%) had solitary lesions and 79.5% patients showed complete clinical response. They noted that as their experience improved, the success rate with ablation improved as well (from 66.6% in the initial series to 89.2% in the last 28 patients). Their published 1- and 3-year survival rates were 87.7% and 62.4%, respectively. In the same year, Xu et al. [56] published a large series on 145 patients evaluating the efficacy and complications of HIFU in patients with HCC. They found that treatment with HIFU resulted in symptom improvement and pain relief in 84.8% patients. They also noted that HIFU caused reduction in the serum AFP value in 71.7% and improved survival. They reported a 2-year survival rate of 80% in patients with stage Ib HCC, 51.4% in stage IIa, and 46.5% in stage IIIa patients.

HIFU has also been compared to other thermal ablation techniques. Recently, a study from China by Chan et al. [57] compared HIFU with RFA in 103 patients with HCC. They noted that the morbidity rate was higher in the HIFU group (7.4% versus 6.5%), but there was not associated mortality. Most of the patients in the HIFU group suffered skin burns or developed pleural effusion. The 3-year overall survival rate associated with HIFU was better than RFA, but statistically insignificant (69.8% and 64.2%, resp.; *P* = 0.19). HIFU has also been evaluated as bridging therapy for patients with HCC awaiting transplant. Cheung et al. [58] evaluated 49 consecutive HCC patients listed for liver transplantation over a period of four years. These patients were treated with different bridging techniques including TACE and HIFU. The authors concluded that HIFU was comparable to other bridging techniques in safety and efficacy in patients with advanced cirrhosis and helped to reduce the drop-out rate amongst liver transplant candidates.

Similar to other newer ablation techniques, HIFU is a very promising noninvasive technology. However, it lacks long-term data on patient safety and survival. Therefore, it is pertinent at this stage to say that further studies are needed before this technique becomes widely acceptable.

#### 2.3.4. Microwave Ablation

MWA is a newer thermal ablation technique that has shown promising long-term outcomes during treatment of hepatic malignancy. This technology relies on generating heat using dielectric hysteresis. The high frequency microwaves (typically 900 to 2500 MHz) cause polarization and rapid oscillation of the intracellular water molecules. This results in transfer of kinetic energy into heat at the given site. The resulting rise in temperature causes coagulative necrosis and ultimately tumor ablation [59].

While the basic working principle of MWA is the same, the commercial devices vary across the world. In Asia and Europe, most of the reported studies have utilized devices working on 2.45 GHz systems (Microtaze system (Nippon Shoji, Osaka, Japan), FORSEA system (Qinghai Microwave Electronic Institute, Nanjing, China), or the Acculis MTA system (Microsulis Medical Ltd, Hampshire, UK)), where as in the USA the published studies have mostly used the 915 MHz models (Evident system (Valleylab/Covidien, Boulder, CO), MicroTherm X-100 system (BSD Medical Corp, Salt Lake City, UT), AveCure system (MedWaves, San Diego, CA), and Certus 140 (Neuwave Medical, Madison, WI)). Most of the 2.45 GHz models are based on a single large diameter antenna, whereas the 915 MHz models can utilize multiple needle antennae powered by separate generators. In the latter, the combining of small diameter antennae allows amplification of the fields and achieves larger ablation volumes for similar sized target lesions using reduced wattage, which potentially decreases the unwanted side effects of over-heated single antennae seen in the former [59, 60].

Due to its initial success as a viable ablation technology, MWA has been well studied and extensively compared with other ablation techniques, especially the RFA. MWA has the advantage that it can be used in multiple ways to achieve tumor ablation including open surgical, laparoscopic, and percutaneous. Secondly, it achieves higher temperatures and larger ablative zones in a shorter time period and has better safety profile as well as less postprocedural pain when compared with RFA. Further, MWA is not affected by the heat sink effect that is typically seen with RFA in lesions close to the large blood vessels. Because microwaves are not insulated by water vapor or charred tissue that is generated during ablation, the ablative zones created by MWA devices are considered more consistent and uniform in character. This is believed to yield greater tumor necrosis and decreases the likelihood of local tumor recurrence [60].

In the last few years, there have been several studies that have looked at the application of MWA in the treatment of hepatic malignancies. These studies have evaluated safety, efficacy, long-term outcomes, and survival as well as compared MWA to other ablation techniques. In one of the earlier studies, Sato et al. [61] evaluated 19 patients with unresectable HCC and advanced cirrhosis. While the tumor size in these patients was very variable, ranging from 0.5 to 9 cm, they were able to successfully ablate all lesions utilizing laparotomy, laparoscopy, or thoracotomy. They found that treatment with MWA led to potentially curative treatment in 14 patients, more than 90% of nodules were completely ablated and 13 patients survived long term. They concluded that MWA was safe and efficient and had the potential to provide long-term survival in certain subsets of patients. More importantly, this technology had opened a new avenue for patients in whom resection was theoretically unimaginable due of their underlying cirrhosis.

Other similar studies were soon published and this started a new era in the advancement of microwave technology. In 2000, Shibata et al. [62] published results of their randomized study comparing MWA with hepatic resection in patients with multiple hepatic metastases from colorectal carcinoma. Fourteen patients were treated with MWA, while 16 patients underwent hepatectomy. Tumors in the MWA group were ablated under ultrasound guidance after laparotomy at an output of 60–100 W for 2–20 minutes. They found that their 1-, 2-, and 3-year survival rates and mean survival times were 71%, 57%, and 14% and 27 months, respectively, in the MWA group, whereas they were 69%, 56%, and 23% and 25 months, respectively, in the hepatectomy group. The difference between these two groups was not statistically significant (*P* = 0.83). Further, the blood loss was significantly less (*P* < 0.05) in the MWA group and 37.5% of patients in the hepatectomy group required blood transfusion. They concluded that MWA was safe, less invasive, and equally effective as hepatic resection in the treatment of multiple hepatic lesions. Another similar study in 2002 by Shibata et al. [63] compared MWA with RFA in 72 patients with 94 HCC nodules. In this randomized study, the authors found that both MWA and RFA had similar therapeutic effects, complication rates, and rates of residual foci of untreated disease. They concluded that, like their previous study, MWA was also equivalent to RFA in its therapeutic benefits.

The experience with MWA has grown significantly over the last 10 years. In 2003, long-term results of percutaneous MWA for the treatment of HCC in a large patient population were published [64]. In this report, all patients were considered nonoperative candidates. There were 208 men and 26 women with a total of 339 nodules. Their tumor size ranged between 1.2–8 cm and the mean follow-up period was 27.9 months. The authors noted that after percutaneous MWA, color Doppler flow signals disappeared in 92.0% of the lesions. On contrast-enhanced CT and MR imaging, no enhancement was apparent in 89.2% and 89.1% of the lesions, respectively. Posttreatment biopsies showed no evidence of surviving tumor tissue in 92.8% of nodules. The 1- and 5-year cumulative survival rates were 92.70% and 56.70%, respectively, and no severe complications were seen. The study concluded that ultrasound guided MWA was safe and effective and resulted in a high percentage of cases without evidence of residual tumor and satisfactory long-term results during treatment of nonresectable HCC.

In 2005, Liang et al. [65] expanded on the previous data published by Dong et al. [64] to provide 8-year follow-up information on prognostic factors and long-term survival in 288 patients with HCC, treated with percutaneous MWA. The reported 1- and 5-year cumulative survival rates amongst all 288 patients were 93% and 51%, respectively. Thirty-two percent of patients died during this period and local recurrence or new tumors were observed in 35% of patients. They found that tumor number, tumor size, and Child-Pugh classification had a significant effect on survival. They concluded that MWA conferred high probability of long-term survival in patients with a single lesion <4 cm and Child-Pugh class A cirrhosis. The same year, a study published from Japan reported 5-year survival rates with laparoscopic MWA (LMWA) in patients with HCC [66]. The authors successfully ablated all lesions laparoscopically. Over the period of study, they found that 12% of patients developed local recurrence, 57% of patients developed distant recurrence, and 21% of patients died from disease. Their 1- and 5-year survival rates were 97% and 43%, respectively. Overall, LMWA was considered safe and effective for treatment of HCC nodules located near the liver surface.

In 2007, Iannitti et al. [67] published the results of the first phase–II trial using MWA in the USA. This study utilized a 915 MHz generator in 87 patients over a period of 2 years. There were 224 hepatic lesions, including both primary HCC and metastatic colorectal tumors. During this study, 45% of ablations were performed via an open procedure, 7% were performed laparoscopically, and 48% were performed percutaneously. At a mean follow-up period of 19 months, they found that local recurrence occurred in 2.7% of tumors; regional recurrence occurred in 43% of patients and 47% of patients were alive with no evidence of disease.

With the safety of this procedure firmly established, authors began to look at other aspects of MWA treatment. In 2008, Shiomi et al. [68] presented results from a study involving 142 patients. They compared thoracoscopically assisted magnetic resonance guided MWA with percutaneous MWA. They evaluated if hepatic tumors located in the subdiaphragmatic area that are difficult to approach by ultrasound could be treated safely. They found that complication rate, recurrence rate, and the length of hospital stay did not differ significantly between the groups. They concluded that MR guided and thoracoscopic assisted MWA was safe for treating subdiaphragmatic lesions. In another study, Yin et al. [69] looked at the feasibility of treating larger tumors with MWA. They treated 109 HCC patients with tumors measuring between 3 and 7 cm with percutaneous RFA and MWA. Over the period of study, they found that there were no treatment-related deaths, the major complication rate was 9.2% and the rate of complete ablation was 92.6%. The 1-, 3-, and 5-year survival rates were 75.8%, 30.9%, and 15.4%, respectively. They concluded that percutaneous MWA and RFA were effective in treating hepatic tumors between 3 and 7 cm, with acceptable local tumor control and long-term outcomes. MWA has been also found to be effective in treating recurrent hepatic lesions. In their study that included 45 patients with recurrent HCC, Itoh et al. [19] found that treatment with MWA was associated with 1- and 3-year recurrence-free survival rates of 41.6% and 8.8%, respectively.

A recent study combined TACE with percutaneous MWA to evaluate response and long-term survival in patients with large unresectable primary HCC (≥ 5.0 cm in diameter) [70]. Amongst the 136 patients that were treated, 80 patients received TACE monotherapy and 56 patients received TACE combined with MWA. These patients were followed for a median time of 41 months (range, 6–96 months). The authors noted that the combination of TACE + MWA statistically improved both the median survival time as well as the 1- and 5-year overall survival rates (25 months, 87.5% and 10.0%, resp.). Another study combined MWA with TACE and sorafenib in patients with recurrent HCC. The authors found that the patients who received sorafenib had better disease control rate as well as survival as compared to patients who did not receive sorafenib (*P* < 0.05) [71]. MWA has also been combined with hepatic resection to improve patient outcomes. A study by Harada compared living donor hepatic transplant (LDLT) with a combination of MWA + hepatic resection (MWA + HR) in a selected group of patients with Child-Pugh class B cirrhosis and HCC [72]. Forty patients underwent LDLT, while 30 patients were treated with MWA + HR. It was found that there was no difference in overall survival between these groups. The 5-year survival rates in the LDLT and MWA + HR groups were 72.6% and 70.4%, respectively. On multivariate analysis, the des-gamma-carboxy prothrombin (DCP) level of more 300 mAU/mL was an independent risk factor for overall survival and recurrence of HCC after LDLT. The study concluded that, in patients with Child-Pugh class B cirrhosis that met the Milan criteria, LDLT offered longer disease-free and overall survival only if DCP levels were less than 300 mAU/mL. In patients with DCP level of more 300 mAU/mL, LDLT was not indicated.

Given the recent surge in studies looking at the use of MWA in many different clinical scenarios, Bala et al. [73] performed a Cochrane review to provide a detailed analysis of the effects of MWA, including its comparison with other ablation methods, with no intervention, and with systemic treatments in patients with liver metastases. The authors conducted a detailed search to identify and include all randomized clinical trials assessing the beneficial and harmful effects of MWA and its comparators, irrespective of the location of the primary tumor. The authors could include only one randomized study in their analysis based on the inclusion criteria of the review. The authors concluded that the evidence is currently insufficient to show whether MWA brings any significant benefit in terms of survival or recurrence compared with conventional surgery for patients with liver metastases from colorectal cancer. Given a paucity of reliable data, the authors recommended that, at present, MWA should not be used instead of conventional surgery in operative candidates outside of randomized clinical trials.

Recent results have generated discussion regarding the “redefined” role of MWA, especially in a broader context of all ablation techniques. There is now a renewed interest to define the role of ablation techniques such as MWA and RFA either alone or in combination with other techniques in patients with hepatic malignancy. While it may be argued that it is still premature to define the role of these ablation techniques until more robust data becomes available, it is prudent that we should keep evaluating these techniques, especially in a selected group of patients in whom the conventional treatment strategies offer limited benefit.  Table 4 provides a summary of studies investigating the role of thermal ablation in patients with hepatic malignancy.

### 2.4. Irreversible Electroporation

IRE is an older technology that has been recently modified for use as an ablation technique for advanced hepatic malignancy. It is based on the principle of creating pores in the lipid bilayer of cell membrane using electric current. Micro- to millisecond electrical pulses at 1,000–3,000 volts are given via needle electrodes, which leads to loss of cellular homeostasis and eventually cell death. The major distinguishing feature of IRE is that it is a nonthermal technology, irreversibly altering the permeability of the tumor cell membrane ultimately leading to apoptosis. Consequently, it has a sparing effect on important structures like bile ducts, blood vessels, and tissue stroma [74]. While a few devices have been developed, the most commonly available commercial device is NanoKnife (AngioDynamics, New York), which utilizes a 2,500 V generator system.

The early clinical experience with IRE has been encouraging and has demonstrated safety and efficacy during ablation of hepatic tumors. Most of the available data is short-term, mostly case reports, case series, or reviews. Charpentier [74] published an initial review looking at safety and efficacy of IRE in different preclinical and clinical studies. They found that IRE was not only safe but also potentially superior to other ablation techniques when utilized to ablate liver tumors abutting major vascular structures or near portal pedicles where heat sink and collateral damage has to be avoided. A retrospective review from Sloan Kettering evaluated IRE specific treatment outcomes, rate of recurrence, and complications in 28 patients who could not be ablated with other techniques due to tumor location. They found that despite close proximity to major structures, IRE was safe and only 3% of patients in their study developed complications. One patient developed intraoperative arrhythmia and another developed postoperative portal vein thrombosis. There were no treatment-associated mortalities. At a median follow-up of 6 months, there was 1 tumor with persistent disease (1.9%) and 3 tumors recurred locally (5.7%) [75]. Working on a similar theme, Cannon et al. [76] performed a prospective study and found 100% initial success with IRE in 44 patients with hepatic tumors over a period of 2 years. They found that 11% of patients in their study developed adverse events; however, all complications were resolved within 30 days. Their local recurrence free survival at 3 and 12 months was 97.4% and 59.5%, respectively. The recurrence rate was found to be higher in patients with tumors over 4 cm (*P*  =  0.178).

While the initial reports are encouraging, a big question is whether this technology will become widely adopted. Is there an associated learning curve and are outcomes related to experience? To answer some of these questions, Philips et al. [77] prospectively evaluated a multi-institutional experience and reviewed the learning curve associated with IRE. They analyzed 150 consecutive patients at seven institutions over two years. The patients were grouped into three groups based on time period to intervention. All three groups were similar with respect to comorbidities and demographics. They found that all three groups had similar complication rates. The morbidity rate was 13.3% and high-grade complications were seen in 4.19%. They found that, with increased experience over a period of time, treatments of larger lesions and lesions with greater vascular involvement were performed without a significant increase in adverse effects or impact on local relapse-free survival. There was a significant improvement with experience, with the learning curve demonstrating proficiency after 5 cases.

Unlike other ablation techniques, it is sometimes challenging to interpret “ablation zones” and the effect of IRE on tissues after treatment. During their review of biliary complications after IRE ablation in hepatic tumors, Silk et al. [78] utilized a combination of CT scan and laboratory values to study the effect of IRE including the evidence of bile duct dilatation, stricture, or leakage. Two out of 11 (18%) patients had persistence of elevated laboratory values, for which a definite cause could not be identified. Another study has looked at the usefulness of contrast-enhanced ultrasound (CEUS) to evaluate posttreatment ablation status using a dynamic recording of the microvascularization [79]. The authors found that CEUS successfully detected reduction in the microcirculation within the ablation lesions following IRE (*P* < 0.001). This reduction of microcirculation in the ablation area was considered as a marker for successful treatment. Unfortunately, at this early stage, it is not known whether this reduction in microcirculation extrapolates into a survival benefit. In another study, IRE was recently compared to RFA with regard to rate of complications and patient tolerance. The authors found that IRE was comparable to RFA with respect to both the patient pain tolerance as well as rate of complications [80].

At present, the treatment of hepatic lesions with IRE appears clinically feasible. The treatment of hepatic tumors can be demanding, especially in locations where conventional ablation techniques are problematic. Long-term outcomes are as yet unavailable which will likely limit the use of IRE on a widespread scale. In the meantime, this technology may benefit selected patients who would otherwise have limited options with currently available ablative treatments.  Table 5 summarizes studies investigating the role of irreversible electroporation in patients with hepatic malignancy.

## 3. Summary

In summary, the clinical management of hepatic malignancy has undergone a sea change in the last decade. Initially, the introduction of tumor ablative techniques was seen as a second line tool, primarily for palliation. However, with advancements in technologies and development of new devices, there has occurred a paradigm shift. The newer technologies like RFA, LITT, HIFU, MWA, and IRE have shifted the focus from simple palliation to potentially curative treatment. The 5-year survival following ablation of patients with HCC as well as some metastatic lesions has improved dramatically, matching results to those obtained by surgical resection or liver transplantation in some studies. This has allowed expansion of the criteria for ablation of hepatic malignancy and led to increased utilization. Currently, patients with large, multiple, or bilateral lesions or lesions previously considered untreatable or unresectable are now being considered for treatment. Ablation is being increasingly combined with other treatments such as TACE, systemic chemotherapy, hepatectomy, or transplant to increase the number of patients who may ultimately benefit from improved survival. While the long-term results regarding the safety and survival benefits of these liver-directed ablative therapies are not substantive at present, we will still likely see the inclusion of some of these within oncology practice guidelines in the near future. Given the significant potential benefits with these technologies, it is important that we strive to evaluate them using appropriate comparative outcomes methodologies.

## Figures and Tables

**Table 1:** Ablation therapies for primary and metastatic liver malignancy.

Chemical ablation	Percutaneous ethanol injection (PEI)
	Percutaneous acetic acid injection (PAI)

Cryoablation	Liquid nitrogen
	Argon
	Nitrous oxide

Thermal ablation	Radiofrequency ablation (RFA)
	Laser-induced thermotherapy (LITT)
	High-intensity focused ultrasound (HIFU)
	Microwave ablation (MWA)

Electroporation	Irreversible electroporation (IRE)

**Table 2:** Summary of studies with chemical ablation (PEI and PAI) in patients with primary and metastatic liver malignancy.

Author(s)	Year	Type of study	Summary and outcome(s)
Shiina et al. [22]	2012	Case series	Aim: to evaluate long-term outcomes in patients treated with PEI for HCC Study data: 685 patients treated with 2147 treatments Conclusions: 5-, 10-, and 20-year survival rates were 49.0%, 17.9%, and 7.2%, respectively; PEI was potentially curative for HCC, resulting in OS of >20 years

Schoppmeyer et al. [26]	2009	Cochrane review	Aim: to evaluate the effects of PEI or PAI in adults with early HCC Study data: three RCTs, 261 patients Conclusions: PEI and PAI do not differ significantly

Riemsma et al. [27]	2013	Cochrane review	Aim: to compare effects of PEI with other treatments in patients with liver metastasis Study data: one RCT, 48 patients Conclusions: addition of PEI to TACE does not confer clear benefit in survival

Cho et al. [28]	2009	Systematic review	Aim: to compare effects of PEI with other treatments in patients with liver metastasis Study data: four RCTs, 652 patients Conclusions: RFA provides improved 3-year survival in patients with HCC, when compared to PEI

Orlando et al. [29]	2009	Meta-analysis	Aim: to compare effects of PEI with RFA in patients with small HCC Study data: five RCTs, 701 patients Conclusions: RFA is superior to PEI with respect to OS, DFS and 1-, 2-, and 3-year survival rates; RFA has better tumor response and smaller risk of local recurrence

Chen et al. [30]	2006	RCT	Aim: to compare PEI with surgical resection in patients with small HCC Study data: 180 patients were randomized into two groups Conclusions: chemical ablation is less invasive and as effective as surgical resection

Koda et al. [31]	2001	RCT	Aim: to compare PEI + TACE with PEI alone in patients with small HCC Study data: 52 patients were randomized into two groups Conclusions: combined PEI + TACE is superior to PEI alone in the treatment of patients with HCC tumors measuring <2 cm in greatest dimension

Giovannini [32]	2002	Review	Aim: to evaluate effects of PEI in patients with liver metastasis Study data: review of multiple studies Conclusions: PEI is effective in only a selected group of patients with small metastases from colorectal, mammary, and endocrine tumors when surgery is contraindicated

DFS: disease-free survival; HCC: hepatocellular carcinoma; OS: overall survival; PEI: percutaneous ethanol injection; PAI: percutaneous acetic acid injection; RFA: radiofrequency ablation; RCT: randomized controlled trial; TACE: Transarterial chemoembolization.

**Table 3:** Summary of studies with cryoablation in patients with primary and metastatic liver malignancy.

Author(s)	Year	Type of study	Summary and outcome(s)
Niu et al. [21]	2007	Case series	Aim: to evaluate long-term outcomes in patients treated with CA for multiple bilobar CRLM Study data: 415 patients; 291 patients treated with HR and 124 treated with HR + CA Conclusions: median OS 32 moths, 5-year survival with HR and HR + CA were 32% and 24%, respectively (*P* = 0.2); overall, long-term survival results of HR + CA for multiple bilobar CRLM are comparable to that of HR alone in selected patients

Zhou and Tang [33]	1998	Case series	Aim: to evaluate the effects of CA with and without other treatments in HCC Study data: 235 patients; 78 patients treated with CA, 58 patients with CA + HALP, 27 patients treated with CA + surgical resection, and 72 patients treated with CA followed by resection of the frozen tumor Conclusions: CA is as effective as other treatments for treating patients with HCC

Wren et al. [34]	1997	Case series	Aim: to evaluate the efficacy of CA in patients with cirrhosis and unresectable HCC Study data: 12 patients (stage II, 2; stage III, 1; stage IVA, 7; stage IVB, 2) Conclusions: CA is feasible and safe and is primarily palliative; it may provide cure in selected patients with lower-stage disease

Haddad et al. [35]	1998	Case series	Aim: to evaluate effects of CA and surgical resection for advanced hepatic tumors Study data: 31 patients Conclusions: CA complements surgical resection but can cause significant morbidity especially in patients with advanced unresectable hepatobiliary tumors

Seifert et al. [36]	2005	Case control study	Aim: to compare morbidity, mortality, recurrence, and survival between CA and liver resection in patients with liver metastases Study data: 223 patients; 168 patients underwent liver resection and 55 patients had CA Conclusions: survival is comparable in selected patients; however, CA is associated with higher rates of hepatic recurrence; CA may not be suitable for patients with resectable disease

Xu et al. [37]	2008	Case series	Aim: to evaluate the safety and efficacy of CA in patients with hepatic colorectal metastases Study data: 326 patients treated with CA for unresectable metastatic lesions Conclusions: CA is safe and complements surgical resection in unresectable tumors

Seifert and Morris [38]	1999	Survey analysis	Aim: to evaluate incidence, morbidity, and mortality during treatment with CA Study data: 134 centers worldwide (44.8% response); 7605 patients; 2173 patients were treated with hepatic CA Conclusions: serious complication like cryoshock develops in 1% of all patients with hepatic CA; cryoshock is responsible for 18.2% of all deaths associated with this treatment

Jungraithmayr et al. [39]	2005	Case series	Aim: to evaluate the effects of CA in patients with primary and secondary liver malignancy Study data: 54 lesions; 19 patients; 17 patients with metastasis, 2 with HCC Conclusions: CA results in high rate of complications and poor long-term tumor control

CA: cryotherapy; HALP: hepatic artery ligation and perfusion; HR: hepatic resection; HCC: hepatocellular carcinoma.

**Table 4:** Summary of studies with thermal ablation in patients with primary and metastatic liver malignancy.

Author(s)	Year	Type of study	Summary and outcome(s)
Solbiati et al. [20]	2012	Case series	Aim: to evaluate long-term outcomes in patients with CRLM treated with RFA and systemic therapy with intention analysis to treat
			Study data: 99 patients with 202 lesions; unresectable lesions or refused surgery Conclusions: 5- and 10-year survivals were 47.8% and 18%, respectively; overall, addition of RFA to chemotherapy achieved local control in large majority of metachronous CRLM

Molinari and Helton [41]	2009	Review	Aim: to compare QOLAS between HR and RFA for HCCs <5 cm in diameter Study data: Markov model generated data from multiple studies Conclusions: HR provides better QOLAS as RFA is associated with increased rate of recurrence that requires multiple sessions; however, for older people, RFA appears to be the best therapeutic option; if the probability of ablation for recurrent disease is equal in the 2 arms, survival benefits of RFA are similar to HR

Schindera et al. [42]	2006	Case series	Aim: to evaluate risks and benefits of RFA in patients with recurrence after HR Study data: 35 patients with 61 tumors Conclusions: RFA is safe and effective in patients with tumors after previous HR; complete ablation was achieved in 88.5% and the 3-year survival was 45%

Chen et al. [43]	2005	Non-RCT	Aim: to compare rates of recurrence and OS in patients with <5 cm HCC treated with HR versus RFA Study data: 44 patients; 40 patients with metastatic lesions and 10 patients with HCC Conclusions: IRE is safe for treating hepatic tumors that are in proximity to vital structures, initial success achieved in 100% of patients; recurrence free survival at 12 months was 59.5%

Lü et al. [44]	2006	RCT	Aim: to compare results of HR versus TA in patients with early HCC Study data: 105 patients; 114 lesions, randomly divided into HR and RFA Conclusions: TA is cheap, minimally invasive and easily accessible; it also achieves equivalent local therapeutic effectiveness and 3-year survival outcome when compared to HR

Ueno et al. [45]	2009	Case series	Aim: to compare long-term outcomes in patients treated with HR versus RFA for small HCC meeting the Milan criteria Study data: 278 patients, divided in three groups: HR, percutaneous RFA, and surgical RFA Conclusions: in patients with small HCCs within the Milan criteria, HR is better in patients with a single tumor and well-preserved liver function. RFA should be chosen for patients with an unresectable single tumor or those with multinodular tumors, regardless of the grade of liver damage; surgical RFA provides better long-term oncological control

Huang et al. [46]	2010	RCT	Aim: to compare RFA with HR for HCC conforming to Milan criteria Study data: 230 patients; divided into 2 groups and followed over 5 years Conclusions: HR provides better OS and RFS and has lower recurrence rates than RFA for patients with HCC meeting the Milan criteria

Yu et al. [47]	2012	Case series	Aim: to evaluate the outcomes in HCC down-staged patients after locoregional treatments Study data: 161 patients; 48 TAE, 7 PEI, 24 RFA, 15 HR, and 34 combination treatments Conclusions: locoregional treatments can successfully downstage patients; these down staged patients show excellent tumor-free and OS rates after transplantation

Mulier et al. [48]	2008	Review	Aim: to review evidence for and against the use of RFA for resectable CRLM Study data: multiple studies Conclusions: for tumors ≤3 cm, local control after RFA is equivalent to that of HR, especially if applied by experienced physicians to nonperivascular tumors

Gillams and Lees [49]	2009	Case series	Aim: to evaluate long-term survival data for patients with CRLM treated with RFA Study data: 309 patients; 617 sessions, 5-year follow-up Conclusions: in selected patients, RFA achieves 5-year survival rate of 24–33%, which is superior to chemotherapy and equivalent to that achieved with HR

Wong et al. [50]	2010	Review	Aim: to evaluate efficacy and utility of RFA in treating CRLM Study data: multiple studies Conclusions: there is a wide variability in the 5-year survival rate (14% to 55%) and local tumor recurrence rate (3.6% to 60%); further studies are therefore needed to better define the role of RFA in patients with CRLM

Ruers et al. [51]	2012	RCT	Aim: to evaluate the benefits of RFA in treating nonresectable CRLM Study data: 119 patients divided into two groups; 59 systemic treatment only and 60 systemic treatment + RFA Conclusions: RFA plus systemic treatment resulted in significant longer PFS in patients with nonresectable CRLM, effect on OS is uncertain

Cirocchi et al. [52]	2012	Cochrane review	Aim: to systematically review the role of RFA in the treatment of CRLM Study data: 1144 records; 18 studies, 1 RCT Conclusions: the PFS was significantly higher in the group that received RFA; however, there was not conclusive information regarding OS

Mack et al. [53]	2001	Case series	Aim: to evaluate clinical outcomes in patients treated with LITT for hepatic metastasis Study data: 705 patients; 1981 lesions treated with 7148 treatment applications over 7 years Conclusions: LITT achieves local tumor destruction using minimally invasive techniques in outpatient setting; the 5-year survival rate is 30%; overall, it results in improved clinical outcomes and survival rates and can be considered a potential alternative to HR

Pech et al. [81]	2007	Case series	Aim: to evaluate safety and efficacy of MR guided LITT in patients with CRLM Study data: 85 patients; 163 nonresectable lesions Conclusions: after 12 months, the local tumor control was 69.4% and median survival was 23 months; overall, LITT was found to be safe and effective for nonresectable CRLM

Eickmeyer et al. [54]	2008	Case series	Aim: to evaluate long-term outcomes with LITT in patients with nonresectable CRLM Study data: 66 patients; 117 nonresectable lesions Conclusions: after 36 months, the survival was 56% in selected patients; overall, LITT has low complication rate and achieves longer survival when compared to patients treated with systemic treatment alone

Zhou et al. [55]	2007	Non-RCT	Aim: to evaluate benefits and adverse effects of TOCE + LITT in patients with HCC Study data: 105 patients divided into two groups; 54 in TOCE + LITT and 51 in TOCE + PEI Conclusions: after 24 months, the survival was significantly better in TOCE + LITT (79.6% versus 60. 8%); overall, TOCE + LITT has low complication rate and achieves good therapeutic effects in patients with HCC

Vogl et al. [23]	2014	Case series	Aim: to evaluate long-term outcomes in patients with CRLM treated with LITT Study data: 594 patients Conclusions: median survival was 25 months; 5-year survival rate was 7.8% and 5-year PFS was 22.3%; overall, LITT is effective, but prognosis is dependent on initial lymph node status, size, and number of hepatic tumors

Ng et al. [24]	2011	Case series	Aim: to evaluate outcome in patients with unresectable HCC treated with HIFU Study data: 594 patients Conclusions: the technique effectiveness rate was 79.5%, which increased with experience; the 3-year survival rate was 62.4%; overall, HIFU is an effective treatment modality for unresectable HCC with a high technique effectiveness rate and favorable survival outcome

Xu et al. [56]	2011	Case series	Aim: to evaluate efficacy and complication rate of HIFU treatment in patients with HCC Study data: 145 patients; single institution Conclusions: symptoms improved in 84.8% patients; the 2-year survival rate was 80% in patients with stage IB, 51.4% in stage IIA, and 46.5% in stage IIIA; overall, HIFU is safe and improves quality of life survival

Chan et al. [57]	2013	Case series	Aim: to evaluate and compare treatment with HIFU with RFA in patients with HCC Study data: 103 patients; 27 treated with HIFU and 76 with RFA Conclusions: 3-year DFS rate was 18.5% in HIFU versus 26.5% in RFA group; 3-year OS was 69.8% in HIFU versus 64.2% in RFA (*P* > 0.05); overall, HIFU has promising results in patients with recurrent HCC, but further evidence is required

Cheung et al. [25]	2013	Case series	Aim: to evaluate HIFU ablation as an effective bridging therapy for patients with HCC Study data: 49 consecutive patients listed for liver transplant based on UCSF criteria Conclusions: 90% patients in HIFU group versus 3% in the TACE group had complete response; 7 patients in the TACE group and no patient in the HIFU group dropped out from the transplant waiting list (*P* = 0.559); overall, HIFU has promising results as a bridging therapy and reduces the drop-out rate of liver transplant candidates

Sato et al. [61]	1996	Case series	Aim: to evaluate safety and efficacy of MWA in HCC Study data: 19 patients with unresectable HCC Conclusions: 73.7% patients achieved potentially curative treatment; overall, MWA is safe, efficacious, and potentially curable in patients with HCC, with advanced liver cirrhosis and multifocal or central tumors

Itoh et al. [19]	2011	Case series	Aim: to evaluate the efficacy of MWA in unresectable HCC Study data: 60 patients; 15 patients with initial HCC and 45 with recurrent HCC Conclusions: 3-year RFS in initial HCC was 36.7% and recurrent HCC was 8.8%; 5-year OS for all patients was 43.1%; overall, MWA is an effective method for treating initial or recurrent unresectable HCC

Shibata et al. [62]	2000	Case series	Aim: to evaluate and compare treatment with MWA with HR in patients with CRLM Study data: 30 resectable patients; 14 treated with MWA and 16 with HR Conclusions: 3-year survival was 27 months in MWA versus 25 months in HR (*P* > 0.05); overall, MWA is equally effective as HR in the treatment of multiple (two to nine) CRLM, whereas its surgical invasiveness is less than that of HR

Dong et al. [64]	2003	Case series	Aim: to evaluate long-term results of percutaneous MWA in patients with HCC Study data: 234 patients with 339 hepatic lesions Conclusions: posttreatment biopsy showed no tumor in 92.8%; 5-year survival rate was 56.7%; percutaneous MWA resulted in a high percentage of cases without evidence of residual tumor and satisfactory long-term results

Liang et al. [65]	2005	Case series	Aim: to evaluate survival and prognostic factors in patients with HCC treated with MWA Study data: 288 patients with 477 lesions Conclusions: 5-year cumulative survival rate was 51%; tumor size, number of nodules, and Child-Pugh classification were prognostic for survival; MWA confers long-term survival in patients with a single lesion <4.0 cm or less and Child-Pugh class A cirrhosis

Seki et al. [66]	2005	Case series	Aim: to evaluate long-term outcomes in patients treated with LMWA for HCC Study data: 68 patients with 71 hepatic lesions Conclusions: 91% effectiveness rate, 5-year survival was 43%; LMWA is a useful modality for treatment of HCC nodules located near the liver surface, and it can be safely performed under direct visual guidance

Iannitti et al. [67]	2007	Non-RCT	Aim: to evaluate outcomes from a clinical trial using MWA in hepatic tumors Study data: 87 patients with 224 tumors; 42 treated with open MWA, 7 with laparoscopic MWA, and 45 with percutaneous MWA Conclusions: local recurrence seen in 2.7% tumors, regional recurrence occurred in 43% patients; at follow-up of 19 months, 47% patients were alive with no evidence of disease; overall, MWA is safe, and clustered antennae confer larger ablation volumes

Shiomi et al. [68]	2008	Case series	Aim: to evaluate and compare magnetic resonance guided treatment of hepatic tumors with percutaneous and thoracoscopic MWA Study data: 142 patients; 73 treated thoracoscopically and 69 percutaneously Conclusions: both techniques are comparable; however, thoracoscopic MWA is minimally invasive and improves targeting of peridiaphragmatic lesions

Yin et al. [69]	2009	Case series	Aim: to evaluate therapeutic efficacy of RFA and MWA in treating HCC >3 cm Study data: 109 patients; 58 were treated with ablation first, while 51 were treated after HR Conclusions: complete ablation rate was 92.6%; local recurrence occurred in 22% and distal recurrence in 53.2%; 5-year survival rate was 15.4%; both RFA and MWA are effective and safe in treating HCC >3 cm (3–7 cm), with acceptable local tumor control and long-term outcomes; completeness of ablation, previous history of treatment, and preablation AFP level were significant prognostic factors

Xu et al. [70]	2013	Case series	Aim: to evaluate long-term outcomes in patients with large HCC (>5 cm) treated with TACE with or without MWA Study data: 136 unresectable patients; 80 treated with TACE and 56 with TACE + MWA Conclusions: 5-year OS rates were 5.0% in the TACE group and 10.0% in the TACE + MWA (*P* < 0.001); TACE + MWA improves survival in patients with large unresectable HCC

Hua and He [71]	2012	Case series	Aim: to evaluate therapeutic efficacy of sorafenib in combination with MWA and TACE in patients with recurrent liver cancer Study data: 90 patients with recurrent HCC; treatment group got sorafenib + MWA + TACE, and the control group received MWA + TACE only Conclusions: treatment group had significant improvement in survival; overall, sorafenib combined with MCT and TACE can improve the disease control rate and prolong the survival in patients with recurrent HCC

Harada et al. [72]	2012	Case series	Aim: to evaluate the results of HR + MWA versus LDLT for HCC in patients with Child-Pugh class B cirrhosis Study data: 70 resectable patients; 30 treated with HR + MWA and 40 with LDLT Conclusions: 5-year survival after HR + MWA was 70.4%, while it was 72.6% after LDLT; DFS was better after LDLT; overall, in preoperative Milan criteria met-cirrhotic patients with Child-Pugh class B, LDLT was associated with longer DFS and OS than HR + MWA

Bala et al. [73]	2013	Cochrane review	Aim: to evaluate beneficial and harmful effects of MWA compared with no intervention, other ablation methods or systemic treatments in patients with liver metastases
			Study data: 40 patients; one RCT Conclusions: there is insufficient evidence to show whether MWA brings any significant benefit in terms of survival or recurrence compared with conventional surgery for patients with CRLM

CRLM: colorectal liver metastases; DFS: disease free survival; HR: hepatic resection; HCC: hepatocellular carcinoma; HIFU: high-intensity focused ultrasound; LMWA: laparoscopic microwave ablation; LITT: laser-induced thermotherapy; LDLT: living donor liver transplantation; MWA: microwave ablation; OS: overall survival; PEI: percutaneous ethanol injection; PFS: progression free survival; QOLAS: quality of life adjusted survival; RFA: radiofrequency ablation; RCT: randomized controlled trial; RFS: recurrence free survival; TA: thermal ablation; TACE: transarterial chemoembolization; TAE: transarterial embolization; TOCE: transarterial oily chemoembolization.

**Table 5:** Summary of studies with irreversible electroporation in patients with primary and metastatic liver malignancy.

Author(s)	Year	Type of study	Summary and outcome(s)
Cheung et al. [25]	2013	Case series	Aim: to evaluate safety, efficacy, and tumor response with IRE in patients with unresectable HCC Study data: 11 patients with 18 tumors Conclusions: 72% tumors were completely ablated, 93% success for lesions ≤3 cm; overall, IRE is a safe and feasible technique for local ablation of HCC, particularly for tumors <3 cm

Charpentier [74]	2012	Review	Aim: to evaluate safety and efficacy of IRE from preclinical and clinical studies Study data: published studies and abstracts Conclusions: IRE is safe and effective and offers advantage over conventional thermal ablation due to absence of heat sink effect and preservation of the acellular elements

Kingham et al. [75]	2012	Case series	Aim: to evaluate safety and short-term outcome with IRE in patients with perivascular malignant liver tumors Study data: 28 patients with 65 tumors; 79% treated with open approach and 21% percutaneous Conclusions: IRE is safe for treatment of perivascular hepatic tumors; overall morbidity was 3%, no mortality, 1.9% rate of tumor persistence and 5.7% rate of tumor recurrence

Cannon et al. [76]	2013	Case series	Aim: to evaluate safety and efficacy of IRE for hepatic tumors Study data: 44 patients; 40 patients with metastatic lesions and 10 patients with HCC Conclusions: IRE is safe for treating hepatic tumors that are in proximity to vital structures, initial success achieved in 100% patients; recurrence free survival at 12 months was 59.5%

Philips et al. [77]	2013	Case series	Aim: to evaluate effects of “learning curve” and experience on outcome with IRE Study data: 150 patients; 3 groups of 50 patients each, based on chronology Conclusions: IRE is a safe and effective alternative to conventional ablation; over time, the proficiency to treat complex lesions improves significantly, with a demonstrable learning curve of at least 5 cases to become proficient

Silk et al. [78]	2014	Case series	Aim: to assess the rate of BC after IRE of hepatic tumors located <1 cm from major bile ducts Study data: 11 patients with 22 hepatic lesions within 1 cm of major hepatic duct Conclusions: IRE offers safe treatment option for centrally located liver tumors with margins adjacent to major bile ducts where thermal ablation techniques are contraindicated

Wiggermann et al. [79]	2014	Case series	Aim: to predict usefulness of CEUS in evaluating ablation zones after treatment with IRE Study data: 20 patients were evaluated before and after treatment with IRE Conclusions: IRE causes significant reduction of microcirculation, which is a marker for successful ablation; CEUS is useful and successfully detects these changes in microcirculation after treatment with IRE

Narayanan et al. [80]	2013	Case series	Aim: to compare postprocedure pain in patients treated with IRE versus RFA for HCC Study data: 43 patients; 21 patients treated with IRE and 22 with RFA Conclusions: IRE is comparable to RFA with respect to postoperative pain

BC: biliary complication; CEUS: contrast-enhanced ultrasound; IRE: irreversible electroporation; HCC: hepatocellular carcinoma; RFA: radiofrequency ablation.
